# Unusual metachronous isolated inguinal lymph node metastasis from adenocarcinoma of the sigmoid colon

**DOI:** 10.1186/1477-7819-9-128

**Published:** 2011-10-14

**Authors:** Adolfo Pisanu, Daniela Deplano, Isabella Reccia, Giuseppina Parodo, Alessandro Uccheddu

**Affiliations:** 1 Department of Surgery, Clinica Chirurgica, University of Cagliari, Cagliari, Italy; 2 Department of Cytomorphology, I Divisione Patologia, University of Cagliari, Cagliari, Italy

**Keywords:** sigmoid carcinoma, inguinal lymph node, unusual metastasis, metachronous metastasis

## Abstract

This study aimed to describe an unusual case of metachronous isolated inguinal lymph nodes metastasis from sigmoid carcinoma. A 62-year-old man was referred to our department because of an obstructing sigmoid carcinoma. Colonoscopy showed the obstructing lesion at 30 cm from the anal verge and abdominal CT revealed a sigmoid lesion infiltrating the left lateral abdominal wall. The patient underwent a colonic resection extended to the abdominal wall. Histology showed an adenocarcinoma of the colon infiltrating the abdominal wall with iuxtacolic nodal involvement. Thirty three months after surgery abdominal CT and PET scan revealed a metastatic left inguinal lymph node involvement. The metastatic lymph node was found strictly adherent to the left iliac-femoral artery and encompassing the origin of the left inferior epigastric artery. Histology showed a metachronous nodal metastasis from colonic adenocarcinoma. Despite metastastic involvement of inguinal lymph node from rectal cancer is a rare but well known clinical entity, to the best of our knowledge, this is the first report of inguinal metastasis from a carcinoma of the left colon. Literature review shows only three other similar reported cases: two cases of inguinal metastasis secondary to adenocarcinoma of the cecum and one case of axillary metastasis from left colonic carcinoma. A metastatic pathway through superficial abdominal wall lymphatic vessels could be possible through the route along the left inferior epigastric artery. The solitary inguinal nodal involvement from rectal carcinoma could have a more favorable prognosis. In the case of nodal metastasis to the body surface lymph nodes from colonic carcinoma, following the small number of such cases reported in the literature, no definitive conclusions can be drawn.

## Background

Inguinal lymph nodes metastasis from colorectal adenocarcinoma is considered as an uncommon clinical occurrence [1]. We report the case of a metachronous isolated inguinal lymph node metastasis from an adenocarcinoma of the sigmoid colon. To the best of our knowledge, this is the first report of inguinal metastasis arising from a carcinoma of the left colon.

## Case presentation

**Figure 1:**
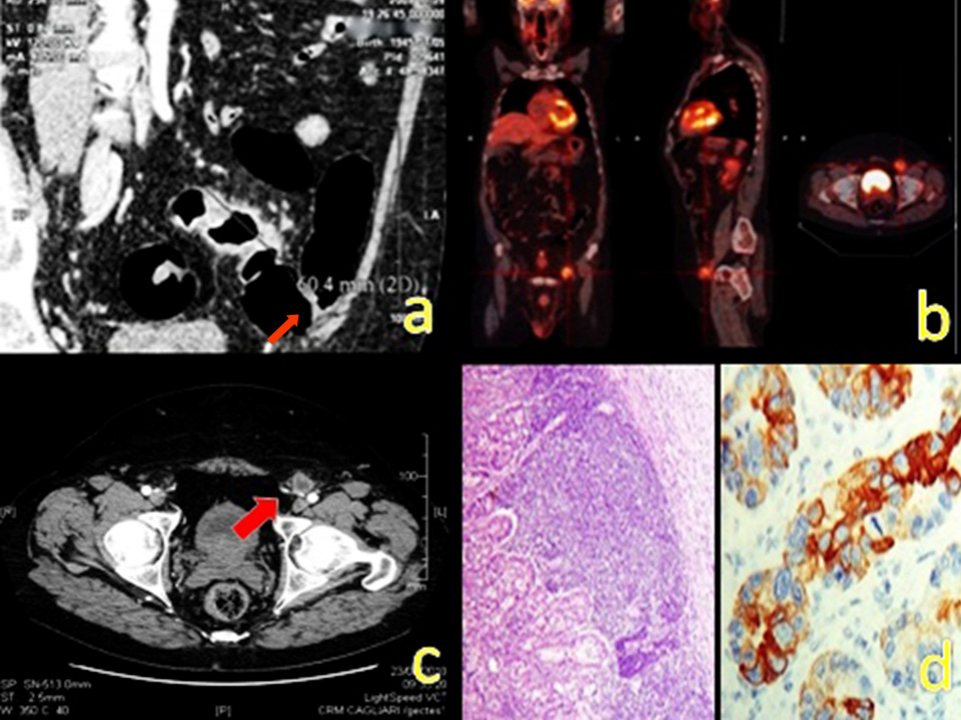
CT-scan showing the obstructing sigmoid carcinoma infiltrating the left lateral abdominal wall (a, thin red arrow), with a 4.5 cm metachronous left inguinal lymph node metastasis at PET-scan (b) and CT-scan (c, thick red arrow), which was histologically  consistent with inguinal nodal metastasis from colonic carcinoma (HE, x 40) and positive expression of cytokeratin 20 at immunohistochemistry (anti-CK20 monoclonal antibody staining, x 100), (d).

In December 2007, a 62-year-old Caucasian man was referred to our surgical department because of an obstructing sigmoid carcinoma. Otherwise, his previous medical history was unremarkable. On examination the abdomen was soft, but distended. Laboratory data on admission were as follows: white blood cell count 82 × 109/L; haemoglobin 11.0 g/dL; and carcinoembryonic antigen (CEA) 10.10 μg/L (normal range <2.50 μg/L). Colonoscopy showed the obstructing lesion located at 30 cm from the anal verge and the enhanced abdominal CT revealed a locally advanced obstructing sigmoid lesion infiltrating the left lateral abdominal wall (Figure 1a, thin red arrow). The patient underwent a colonic resection extended to the infiltrated abdominal wall. Postoperative hospital stay was uneventful. Histology showed an adenocarcinoma of the colon infiltrating the abdominal wall with iuxtacolic lymph node involvement (pT4N1M0). Afterward, the patient was treated with adjuvant chemotherapy which was performed using the FOLFOX regimen (Folinic acid: leucovorin; 5-fluorouracil: 5-FU; oxaliplatin) for 12 cycles over the 6 months after the operation. The patient entered a scheduled clinical and instrumental follow up program which included: regular physical examinations and CEA tests every 3 months, colonoscopy after 6 months and chest/abdominal/pelvic CT on yearly basis. The abdominal CT at 24 months showed no recurrence and CEA level was within normal range. Thirty three months after the operation, CEA level was 6.86 μg/L. Abdominal CT and positron emission tomography (PET) revealed a metastatic left inguinal lymph node involvement (Figure 1b-c, thick red arrow). The patient noticed a mild tenderness in the left inguinal region without swelling. On admission, physical examination showed an induration with mild tenderness in the left inguinal region but there was no clinical evidence of a mass as the metastatic lymph node was deeply located under the left inguinal ligament. Physical examination and diagnostic work up excluded any concurrent malignancies both in the perineal region and in the scrotum. After the diagnosis of metachronous inguinal lymph node metastasis was made, a colonoscopy was performed showing normal findings in the colon and at the anastomotic site which was found at 13 cm from the anal verge. The patient was scheduled for an inguinal lymphadenectomy. The metastatic lymph node of 4.5 cm in diameter was found under the left inguinal ligament strictly adherent to the iliac-femoral artery, encompassing the origin of the left inferior epigastric artery. Postoperative hospital stay was uneventful. Histopathological findings were consistent with the diagnosis of metachronous nodal metastasis from colonic adenocarcinoma (Figure 1d, HE, × 40). Immunohistochemistry demonstrated the positive expression of cytokeratin 20 (CK20+) and the absence of cytokeratin 7 (CK7-) substantiating its gastrointestinal origin (Figure 1d, anti-CK20 monoclonal antibody staining, × 100). After recovery the patient was once more subjected to adjuvant chemotherapy. Inguinal lymph node recurrence was regarded as systemic disease by the oncologists and the FOLFIRI regimen (Folinic acid: leucovirin; 5-fluorouracil: 5-FU; irinotecan) was started. This cycle was repeated every two weeks for 6 months. Cetuximab, a monoclonal antibody to epidermal growth factor receptor, was added to FOLFIRI. No major side effects of chemotherapy were observed and eight months after lymph node removal the patient is surviving apparently disease free.

## Discussion

Metastastic involvement of inguinal lymph node from rectal cancer is a rare but well known clinical entity [1]. However, detecting inguinal metastasis from a colonic carcinoma is extremely uncommon even in patients with advanced cancer stage. We have described the first reported case in which an isolated inguinal lymph node metastasis originated from an adenocarcinoma of the sigmoid colon infiltrating the abdominal wall. The most interesting aspect of the present case is the atypical lymphatic route the sigmoid carcinoma followed to metastasize to the inguinal lymph nodes. The usual pattern of regional lymph nodes metastasis in colorectal carcinoma follows the vascular distribution in the mesocolon. Tumors originating from the cecum spread to the ileocolic nodes, whereas tumors of the ascending and the proximal transverse colon drain in lymph nodes of the right colic and middle colic arteries reaching the superior mesenteric artery lymph nodes. Tumors originating from the descending colon spread to the left colic artery nodes, whereas those of the sigmoid colon reach the sigmoid arteries nodes ending in the inferior mesenteric artery lymph nodes. Both the inferior and the superior mesenteric arteries nodes belong to the pre-aortic nodes. Tumors in the rectum can spread by two different routes. The lymphatic drainage of tumors of the upper rectum reaches the inferior mesenteric artery lymph nodes via the superior rectal arteries. Metastases of tumors originating from the lower rectum reach the internal iliac nodes by following the pathway of the middle and the inferior rectal arteries and then the common iliac and the para-aortic nodes. The lymphatic drainage of all colorectal tumors ends in the cisterna chyli that drains into the thoracic duct. Tumors of the anal region spread to the superficial inguinal lymph nodes ascending along the femoral vessels to the deep inguinal nodes and along the iliac vessels to the para-aortic nodes [2.3].

In the present case, as the sigmoid carcinoma invaded the abdominal wall, the most likely hypothesis is that the tumor metastasized to the deep inguinal lymph nodes through a lymphatic pathway along the left inferior epigastric artery being its origin encompassed by the tumor [4]. Moreover, after radical surgery for carcinoma of the rectum with interruption of the normal pathway of lymphatic drainage, recurrent disease may find an alternative retrograde route to the superficial and deep inguinal nodes [2,5]

Literature review shows only three other similar reported cases of colonic carcinoma with solitary nodal metastasis to the body surface lymph nodes: one case of metastatic inguinal lymphadenopathy secondary to adenocarcinoma of the cecum [6]; another case of isolated right external iliac lymph node recurrence from a primary cecum carcinoma [4] and the axillary localization of nodal metastasis from left colonic carcinoma [5]

In the case of metachronous isolated right external iliac lymph node recurrence from cecum carcinoma reported by Uehara *et al*., tumor invasion into the abdominal wall was suspected macroscopically at the time of the first operation. In this case the authors supposed a lymphatic pathway along the right inferior epigastric artery to explain this unusual metastatic occurrence [4]. A similar lymphatic pathway could be possible in the case reported by Hakeem *et al*. of a synchronous contralateral inguinal lymph nodes metastasis from an adenocarcinoma of the cecum with infiltration into the adjoining peritoneal fat [6]. In the case of left metachronous axillary lymph node metastasis from an obstructing and invasive carcinoma of the left colon, the authors hypostasized a metastatic lane through superficial abdominal lymphatic vessels, the periumbilical ones, then to the parasternal lymph nodes and then to the internal mammary nodes, finally reaching the axillary ones [5].

## Conclusions

Lymphadenectomy followed by chemotherapy seems to be the preferred treatment of isolated inguinal metastasis from rectal carcinoma, and the solitary nodal involvement could have a more favourable prognosis [7]. However, in the case of nodal metastasis to the body surface lymph nodes arising from colonic carcinoma, following the small number of such cases reported in the literature, no definitive conclusions can be drawn.

## List of abbreviations

CEA: carcinoembryonic antigen; CK: cytokeratin; CT: computer tomography; FOLFOX: Folinic acid; 5-fluorouracil; oxaliplatin; FOLFIRI: Folinic acid; 5-fluorouracil; irinotecan; 5-FU: 5-fluorouracil; HE: hematoxylin & eosin; PET: positron emission tomography.

## Consent

Written informed consent was obtained from the patient for publication of this case report and any accompanying images. A copy of the written consent is available for review by the Editor-in-Chief of this journal

## Competing interests

The authors declare that they have no competing interests.

## Authors' contributions

AP: conception and study design and manuscript writing and drafting the article and revising for intellectual content. DD and IR: acquisition of data and manuscript writing and revising for intellectual content. GP: revising the manuscript for intellectual content and supportive work. AU: revising the manuscript for important intellectual content. All authors read and approved the final manuscript.
